# Primary intraosseous osteolytic meningioma of the skull: a case report

**DOI:** 10.1186/1757-1626-2-7413

**Published:** 2009-05-18

**Authors:** Abdolreza Sheikhrezaie, Ali Tayebi Meybodi, Mohammad Hashemi, Sajad Shafiee

**Affiliations:** Department of Neurosurgery, Imam Khomeini Hospital, Tehran University of Medical SciencesTehran 14197Iran

## Abstract

Primary intraosseous meningiomas are uncommon. The osteolytic variants of these tumors are even rarer. When one reviews a bony skull lesion, the differential diagnosis is very wide and includes both malignant and benign diseases. In case of primary skull meningioma, correct diagnosis and total resection of the lesion ensures curative therapy. Presented is a case of osteolytic skull lesion not invading the dura that was proved to be a fibrillary meningioma with regions of syncitial pattern.

## Introduction

Primary meningiomas of the skull without involvement of the dura and intracranial cavity comprise an uncommon entity. These lesions often appear as hard osteoblastic tumors which appear as hyperdense areas of the calvarial bones in x-ray studies [1]. They are also different from the so-called *en plaque* or “carpet” meningiomas in that the latter involve the dura and intracranial cavity. Of a rarer prevalence are lytic skull meningiomas that are not readily suspected because of their radiological appearance; rather they are investigated first for a primary source of malignancy elsewhere in the body or are thought to be other lytic lesions of the skull. Yet, osteolytic meningiomas, although rare, should be considered when approaching to a lytic skull lesion since their natural history and treatment outcome are much different from other more common diagnoses in the skull such as metastatic cancer.

## Case presentation

A 62-year-old right-handed male Iranian farmer presented with a soft enlarging mass in the left fronto-parietal region. The mass had come to the patient's attention over last 8 months but was ignored until it was of a considerable size. Its largest diameter was about 9 centimeters and it was a fluctuating mass. The patient had undergone scalp radiotherapy for treatment of ringworm about 45 years ago. He had no remarkable medical history. Drug history was negative and he did not smoke. Skull x-rays and head computed tomogram (CT) showed a lytic lesion of the skull in the left fronto-parietal region.

An extensive physical examination and laboratory/radiologic work-up including a whole body radionuclide scan of bone showed no other lesion elsewhere in the body. Levels of tumor markers such as prostatic specific antigen (PSA) and evaluation of chest and abdomen were normal.

**Figure 1.**
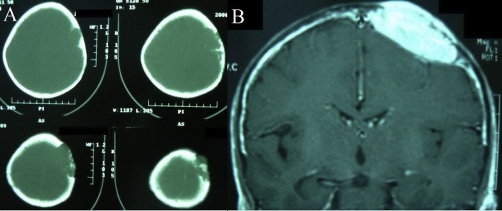
**A.** Axial bone window of the computed tomogram of the presented case showing a destructive osteolytic mass lesion in the left frontoparietal region. **B.** contrast enhanced coronal magnetic resonance imaging of the lesion, showing enhancement of the mass and no involvement of the intradural space.

Magnetic resonance imaging of head showed an extradural intradiploic enhancing mass lesion without intracranial extension (Figure 1).

The patient underwent surgery for resection of the mass lesion. Skin was easily reflected and the mass was not adhered to it. The lesion appeared as a soft lobulated gray mass that had destroyed the calvarial bone. It was easily suctioned and had extension to the peripheral diploe. The infiltrated bone around the lesion was resected so that a rim of healthy bone with normal strength was reached. The dura was intact and the lesion was easily peeled off.

**Figure 2.**
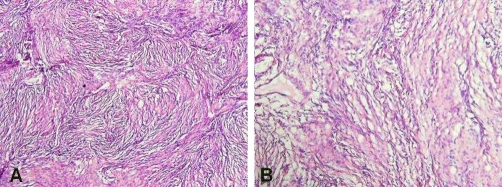
Photomicrographs of **(A)** low and **(B)** high power of the specimen compatible with fibrillary meningioma and scant areas of syncitial pattern (H&E).

Pathologic examination of the lesion revealed fibrillary meningioma with areas of syncithial differentiation (Figure 2). Regarding the calvarial defect after tumor resection, a cranioplasty procedure was planned to be performed in a later session.

## Discussion

Extradural meningiomas constitute 1-2% of all meningiomas [2]. The term “primary extradural meningiomas” differentiates tumors that arise separately from the dura from those that originate in the dura but have an extracranial extension [1]. Lang et al have proposed a classification system for these tumors [3]: tumors that are purely extra-calvarial are type I, purely calvarial tumors are type II, and calvarial tumors with extracranial extension are type III.

Primary intraosseous meningiomas are usually of osteoblastic subtype. More rarely, these lesions may present as an osteolytic skull lesion [1]. The differential diagnosis of an osteolytic lesion of the skull includes chondroma, epidermoid cyst, osteogenic sarcoma, myeloma, metastatic cancer, or fibrous dysplasia [4]. Due to benignity of meningiomas, and hence their different natural history, the intraosseous meningiomas should be considered in evaluation of a lytic skull lesion since definite treatment (i.e., complete surgical resection) is available. If there is doubt about complete resection, the lesion should be followed with appropriate imaging studies [5].

## List of abbreviations

CT: Computed tomography
PSA: Prostatic specific antigen
